# Comparison of the Glucocorticoid Concentrations between Three Species of *Lemuridae* Kept in a Temporary Housing Facility

**DOI:** 10.3390/ani10061013

**Published:** 2020-06-10

**Authors:** Martina Volfova, Zuzana Machovcova, Eva Voslarova, Iveta Bedanova, Vladimir Vecerek

**Affiliations:** Department of Animal Protection and Welfare and Veterinary Public Health, Faculty of Veterinary Hygiene and Ecology, University of Veterinary and Pharmaceutical Sciences Brno, 612 42 Brno, Czech Republic; machovcovaz@vfu.cz (Z.M.); voslarovae@vfu.cz (E.V.); bedanovai@vfu.cz (I.B.); vecerekv@vfu.cz (V.V.)

**Keywords:** zoo, non-invasive, faecal analysis, glucocorticoid metabolites, enzyme immunoassay

## Abstract

**Simple Summary:**

Lemurs kept in captivity are constantly influenced by a number of factors that affect their welfare. All lemur species are classified as endangered species according to the International Union for Conservation of Nature (IUCN) Red List and their population in the wild is decreasing. It is therefore useful to improve the methods for assessing the level of stress in individual lemurs in captivity. In this study, we compared the changes in glucocorticoid concentrations in three species of *Lemuridae* in response to various types of potential stressors during their stay in a temporary housing facility. The glucocorticoid levels were specifically monitored for ring-tailed lemurs (*Lemur catta*), white-headed lemurs (*Eulemur albifrons*) and collared brown lemurs (*Eulemur collaris*).

**Abstract:**

We compared the glucocorticoid concentrations in response to various types of potential stressors present during standard operation of a temporary housing facility between three species, namely, ring-tailed lemurs, collared brown lemurs and white-headed lemurs. The levels of faecal glucocorticoid metabolites (FGMs) were measured non-invasively on a daily basis during a 30-day period. A total of 510 faecal samples were collected. Concentrations of immunoreactive glucocorticoid hormone metabolites were measured in the obtained extracts by using an enzyme immunoassay. The polyclonal antibodies used in this assay were directed against the metabolite 11-oxo-etiocholanolone I. We found all three monitored lemur species to respond to specific potentially stressful situations by increasing (*p* < 0.05) the FGM levels within one to two days after the event. Although housed in the same room, differences in response to potentially stressful situations were found in white-headed lemurs compared to ring-tailed lemurs. Increased mean levels of the FGMs were found more frequently in white-headed lemurs than in ring-tailed lemurs. The results suggest that this species may be more sensitive to changes in its surroundings. In general, the levels of the FGMs showed a similar pattern during 30 days of monitoring suggesting that all groups of lemurs responded in a similar manner to the same events. However, we recorded the differences in the absolute values of glucocorticoid concentrations between the monitored species likely due to the differences in sex ratios in the groups and presence of lactating females in the ring-tailed lemurs.

## 1. Introduction

Animals kept in zoos are constantly influenced by a number of factors that affect their welfare. In order to ensure the welfare of captive animals, it is essential to avoid excessive and, in particular, prolonged stress [1]. Even general husbandry tasks, such as handling, transport and veterinary treatment can be potential stressors [2]. These may cause a stress response, especially in more sensitive individuals [2]. 

Several methods are commonly used to evaluate stress response. They are largely based on the determination of glucocorticoid hormone levels of cortisol and corticosterone or their metabolites [3,4]. A commonly used method for determining the glucocorticoid concentration is to test the blood of the animal. However, in some zoos and in wild animals, the possibility of blood collection is limited and sometimes cannot be performed at all [5,6]. Blood collection brings about many risks, such as injuries caused during catching or sedation of the animal [6]. A more suitable technique is a non-invasive method of assessing stress levels [6], such as the determination of glucocorticoid hormone levels from urine, saliva, fur, milk or animal faeces. This non-invasive assessment of animal welfare is a practical method used for an increasing number of species not only of wild animals, but also of laboratory or farm animals and pets [7]. Collection of faeces seems to be the most appropriate in cases of stress assessment in exotic animals that are not used to handling by humans [8].

Reactions to stressors often vary among different animal species [7,9,10]. Detailed knowledge of the individual species helps to understand their natural behaviour and responses to stressful situations [11]. According to Mason [12], different animal species perceive the conditions of captivity differently. For example, small primates were more stressed by the presence of zoo visitors than larger primates. Differences between ground and tree species were also observed [12]. Weingrill et al. [13] compared the glucocorticoid hormone levels as stress markers in Bornean (*Pongo pygmaeus*) and Sumatran orangutans (*Pongo abelii*). Higher levels of glucocorticoids in the Bornean orangutans were predicted, due to the much more solitary lifestyle and higher frequency of male aggression directed towards females in this species in the wild, which was subsequently confirmed [13]. Furthermore, Cocks [14] found that the hybrids of Bornean and Sumatran orangutans were less resistant to stress than both subspecies, suggesting that the lower survival rate of hybrid orangutans is due to reduced fitness associated with hybridization. Given the variability in species’ stress responses and the variety of animals held under human care, understanding species-specific trends in stress responses to daily stressors in captivity can be useful for guiding management practices.

To achieve reliable results via non-invasive monitoring in individual species, knowledge of its metabolism and the mechanism of glucocorticoid hormone excretion is essential [1]. According to Möstl and Palme [1], the concentration of cortisol metabolites in faecal samples reflects the stress-induced glucocorticoid levels with a delay. The species-specific time delay depends on the metabolism and gut passage time of the animal [9]. Steroids are metabolised in the liver and excreted via the bile into the gut. Therefore, measured concentrations of faecal glucocorticoid metabolites (FGMs) reflect an event occurring a certain time ago. This lag time may range from less than 30 min to more than one day, depending on the species and its activity rhythms [5,7,9]. Cambell et al. [15] reported, for instance, that the digestive tract structure varied among different lemurs. They generally have a simple stomach with a slightly prolonged small intestine, sac-like appendix and large intestine of varying length. Black-and-white-ruffed lemurs (*Varecia variegata*) are reported to have a fast passage of the digestive tract (2 to 4 h). Given the very similar structure of the digestive tract in ring-tailed lemurs (*Lemur catta*) [15], the time of intestinal passage will probably be similar. It can be assumed that for similar species belonging to the same family as in this case, after the method has been validated for one species, the same method can be used for a related species, as demonstrated by the following studies evaluating the stress response in lemurs. Balestri et al. [16] compared the faecal glucocorticoid metabolite (FGM) levels among a group of collared brown lemurs (*Eulemur collaris*) living in fragments of forests with a group living in better preserved areas. For the determination of glucocorticoid hormones in the faeces, a successfully validated analysis for this species targeted against the metabolite 11-oxo-etiocholanolone was chosen. Similarly, glucocorticoid levels in faeces during the reproduction season were analysed in red-fronted lemurs (*Eulemur fulvus rufus*) [17] using the analysis targeting the metabolite 11-oxo-etiocholanolone. The suitability of the analysis of the metabolite 11-oxo-etiocholanolone has also been confirmed by studies of the stress level in ring-tailed lemurs and black-and-white-ruffed lemurs by non-invasive methods [18,19].

*Lemuridae* are generally classified as endangered species according to the International Union for Conservation of Nature (IUCN) Red List and their populations in the wild are decreasing although they are frequently kept in captivity. In order to ensure their stable population, health and welfare in captivity, it is important to reduce the negative effects of stress. A commonly measured endocrine response to stress is the secretion of glucocorticoids [20]. Consequently, there is a need for validation of the methods for assessing the changes in glucocorticoid concentrations in the individual animals non-invasively [1,10].

The aim of this study was to compare the changes in glucocorticoid concentrations in response to various types of potential stressors occurring during a 30-day period in a temporary housing facility in the three selected species of *Lemuridae*, namely ring-tailed lemurs (*Lemur catta*), white-headed lemurs (*Eulemur albifrons*) and collared brown lemurs (*Eulemur collaris*). Given their small body size and fast metabolism, changes in FGMs were expected to be measurable within one to two days after exposure to a stressor [1,10] as confirmed by several studies assessing changes in FGM concentrations resulting from stress, for example, in zoo-living orangutans (*Pongo* spp.) [13], common marmoset (*Callithrix jacchus*), long-tailed macaque (*Macaca fascicularis*), Barbary macaque (*Macaca sylvanus*), chimpanzee (*Pan troglodytes*) and gorilla (*Gorilla gorilla*) [21], Western lowland gorilla (*Gorilla gorilla gorilla*) [22], pileated gibbons (*Hylobates pileatus*) [23], wild gray mouse lemurs [24], ring-tailed lemurs (*Lemur catta*) [18] and black-and-white ruffed lemurs (*Varecia variegata*) [19]. 

## 2. Materials and Methods 

### 2.1. Ethics Statement

This study was carried out in strict accordance with the Directive 2010/63/EU of the European Parliament and of the Council of 22 September 2010 on the protection of animals used for scientific purposes and Czech national legislation (i.e., Act no. 246/1992 Coll.) on the protection of animals against cruelty, as amended. All samples were collected non-invasively and animals did not undergo any experimental procedures. Sampling was carried out during temporary housing of three species of *Lemuridae* in an approved holding facility. Since only non-experimental clinical veterinary practices were performed and no handling of animals related to research was carried out, a formal ethics approval from the Animal Welfare Body of the University of Veterinary and Pharmaceutical Sciences Brno with regard to the EU Directive 2010/63/EU was not required.

### 2.2. Study Subjects and Housing

For the analysis, faecal samples from 9 ring-tailed lemurs, 6 collared brown lemurs and 2 white-headed lemurs were collected for a period of 30 days. The lemurs originated from European zoos and were temporarily housed in a holding facility in Poštovice in the Czech Republic operated by a certified international transportation company, The Nature Resource Network, that was in charge of their transport to other zoos. Details on individual animals monitored in the study are given in Table 1. 

All lemurs were fed primate dry formula (pellets), vegetables (carrot, celery, cucumber, lettuce, red beat, onion, boiled rise, tomatoes) and fruits (banana, apple, peach, pineapple, grapes, kiwi) two times in a day, with green leaves, and with cheese and boiled egg once a week. Drinking water was available ad libitum. The lemurs were housed in indoor enclosures (natural light provided from windows) without access to an outdoor area. Changes in the groups during the monitored period are described below. 

**Figure 1:**
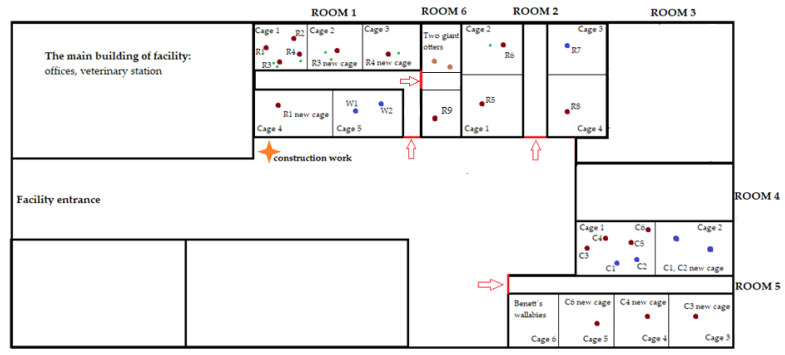
Scheme of floor plan of the facility and relocation of individuals. Red points indicate females; blue points indicate males; green points indicate offspring; red arrows indicate the entrance to the rooms. R1–R9 indicate ring-tailed lemurs 1–9; W1–W2 indicate white-headed lemurs 1–2; C1–C6 indicate collared brown lemurs 1–6.

In ring-tailed lemurs, 9 adult individuals were monitored (8 females and 1 male). There were 4 offspring in the ring-tailed lemurs but they were not included in the analysis. Four adult females (R1–R4) were housed in cage 1 in room 1. Two of them had offspring (R3 had two offspring, R4 had one offspring). The two lactating females (R3 and R4) together with their offspring were separated on day 16 of monitoring to another cage in the same room due to their agonistic behaviour. Female R3 with her offspring was housed in cage 2 and female R4 with her offspring was housed in cage 3. The remaining two adult females (R1 and R2) were left in cage 1. Subsequently, female R1 was isolated in a separate cage 4 in the same room due to injury on the 23rd day of observation. Another four adult ring-tailed lemurs (R5–R8) were housed in room 2. Each adult was individually housed in separate cages, except for female R6, who was co-housed with her offspring. The first day of monitoring one more adult female ring-tailed lemur (R9) arrived at the facility and was placed in a cage in room 6. Rooms 1 and 6 were interconnected. In white-headed lemurs, 2 individuals (both males, W1 and W2) were housed together throughout the whole monitoring period in room 1 in cage 5. In collared brown lemurs, 6 individuals were assessed: 2 males (C1 and C2) and 4 females (C3–C6). Originally, they were housed together in cage 1 in room 5. During the monitored period, however, they were divided into smaller groups due to mutual attacks and relocated to different cages within the same room (room 5). Namely, on day 16 two males (C1 and C2) were relocated to cage 2, on day 18 one female (C3) was relocated to cage 3, on day 23 one female (C4) was relocated to cage 4 and on day 29 one female (C6) was relocated to cage 5. While the ring-tailed lemurs and white-headed lemurs were housed in the same room, the collared brown lemurs were kept on the other side of the facility. A scheme of the floor plan of the facility and relocation of individuals is provided in Figure 1. All lemurs had visual, olfactory and auditory contact with other animals of the same species during the entire monitored period. All animals were clinically healthy without any signs of health problems or behavioural disorders.

### 2.3. Faecal Sampling and Analysis

The monitoring was carried out for 30 days when three lemur species were kept in the facility. Lemurs were exposed to the same husbandry activities (cage cleaning, feeding and watering, visual control of the animals by caretakers). All activities related to the operation of the facility were recorded (e.g., construction work, increased noise, newly incoming animals, transfers within the facility, lactating animals, etc.) by the caretakers during the monitored period in a systematic way with the use of pre-prepared sheets. The faecal samples for the determination of FGMs were collected from the floor once a day between 8:00–12:00 a.m. from each animal, ensuring that they were not contaminated with urine, water or other material. A total of 510 faecal samples were taken. There was no variability in the number of samples taken from different animals. The collected samples were put in plastic sealable bags within 1 h of defecation, labelled and immediately frozen at −20 °C. The subsequent sample processing including extraction was performed at the University of Veterinary Medicine in Vienna, Austria. The samples were thawed, individually homogenised and then successively put into test tubes at weights between 0.480 and 0.520 g. Afterwards 1 mL of distilled water and 4 mL of 100% methanol were added to the weighed samples. Each sample with water and methanol was stirred on a shaker for 30 min and then centrifuged at 4 °C (3750× *g*, 10 min). The detailed extraction process is described in other methodological publications [1,9,25]. Concentrations of immunoreactive glucocorticoid hormone metabolites were measured in the obtained extracts by using an enzyme immunoassay. The polyclonal antibodies used in this assay were directed against the metabolite 11-oxo-etiocholanolone I (laboratory code: 72a, first described by Palme and Möstl [26]), based on previously conducted studies. 

### 2.4. Statistical Analysis

The results were statistically evaluated using the statistical program Unistat 5.6 (Unistat Ltd., London, UK). Data normality was tested using the Shapiro–Wilk test [27]. Since the data were not distributed over the Gaussian curve, the statistical significance of the FGM level differences between the monitored lemur species during the 30 days of observation was determined on each day using a non-parametric Kruskal-Wallis ANOVA test [27] and subsequently nonparametric multiple comparisons with t-distribution using rank sums [27]. For each lemur species, the mean value of the FGMs measured on the first day of the observation was also compared to each subsequent day to determine statistical significance of the differences in the FGM levels by means of the Friedman ANOVA test [27] and subsequently the Dunnett test using rank sums [27]. A value of *p* < 0.05 was considered significant. The first day of sample collection was the day when FGM levels were expected not to be affected by stress, not even in those individuals who arrived on the first day of observation due to a time delay in excretion of FGMs [1]. Therefore, the data from the first day were used as the reference point. FGM levels measured during the monitored period were compared with the timeline of events recorded in the facility in order to determine possible factors eliciting the increase in FGM levels. 

## 3. Results

### 3.1. Ring-Tailed Lemurs

Records on daily events showed a variety of activities occurring in and outside the rooms where lemurs were kept during a 30-day period of monitoring. In room 1, besides daily routine (e.g., cleaning cages, feeding, visual control of animals), noise from construction work was also recorded. Namely, on the 13th day of the monitored period, all-day construction work was carried out from the other side of the room where the ring-tailed lemurs were housed. The construction work was also performed on day 14. Furthermore, some females originally housed in one group in the same cage were moved to separate cages. On day 16, two females (R3 and R4) with offspring were relocated into two separate cages and on day 23, one female (R1) was moved due to an injury to a separate cage, but all stayed in the same room. On day 29, two giant otters (*Pteronura brasiliensis*) were relocated to room 6 (between room 1 and 2, where ring-tailed lemurs and white-headed lemurs were housed). The lemurs had auditory and olfactory contact with these two otters. 

**Figure 2:**
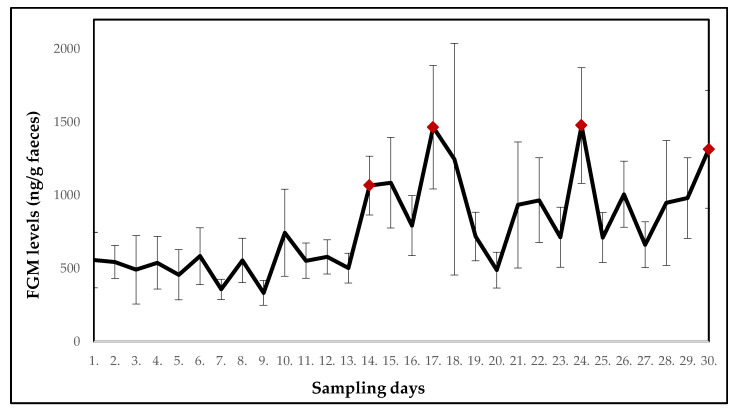
Mean concentrations of the faecal glucocorticoid metabolites (FGMs) in ring-tailed lemurs; significant increases compared to the first day of sampling are indicated. Red points indicate a significant increase in FGM levels.

Fluctuations in the mean FGM levels in ring-tailed lemurs (*n* = 9) over a 30-day period of monitoring are shown in Figure 2 (F_(29.232)_ = 2.3765). On the 14th day of the monitored period, a significant increase (*p* < 0.05) in the mean FGM levels was found. The mean FGM levels also increased on day 17 (*p* < 0.01) and day 24 (*p* < 0.05). Subsequently, on the last day of monitoring, a significant increase in the mean FGM values (*p* < 0.01) was also observed. On other days, the FGM levels did not significantly differ from the first day. 

The mean concentrations of FGMs and potentially stressful events for each day of monitoring are shown in Table 2.

### 3.2. White-Headed Lemurs

**Figure 3:**
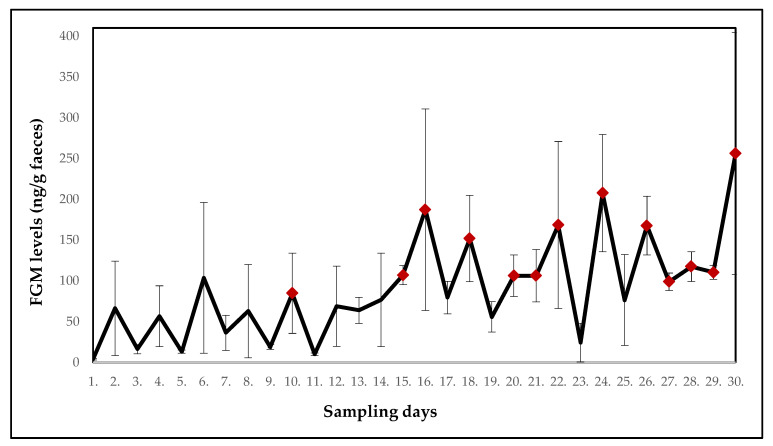
Mean concentrations of the faecal glucocorticoid metabolites (FGMs) in white-headed lemurs; significant increases compared to the first day of sampling are indicated. Red points indicate a significant increase in FGM levels.

Fluctuations in the mean FGM levels in white-headed lemurs (*n* = 2) housed together in one cage located in the same room as the ring-tailed lemurs over a 30-day period of monitoring are shown in Figure 3 (F_(29.29)_ = 1.6832). A significant increase (*p* < 0.05) in the mean FGM levels was found on days 10, 15, 16, 18, 24, 26, 29 and 30 in comparison with the first day of sampling. 

Table 3 summarises the mean levels of the FGMs in the white-headed lemurs and potentially stressful events for each day of monitoring.

### 3.3. Collared Brown Lemurs

They were housed in a different room than the other two lemur species. All of them arrived at the facility on the first day of monitoring and were initially placed together in cage 1 in room 5. During the monitored period, however, some individuals had to be moved due to mutual attacks as described in Section 2. In addition to the changes in the collared brown lemur group, Bennett’s wallabies (*Macropus rufogriseus*) arrived on the third day after the beginning of the observation and they were temporarily placed in the same room as the collared brown lemurs. 

**Figure 4:**
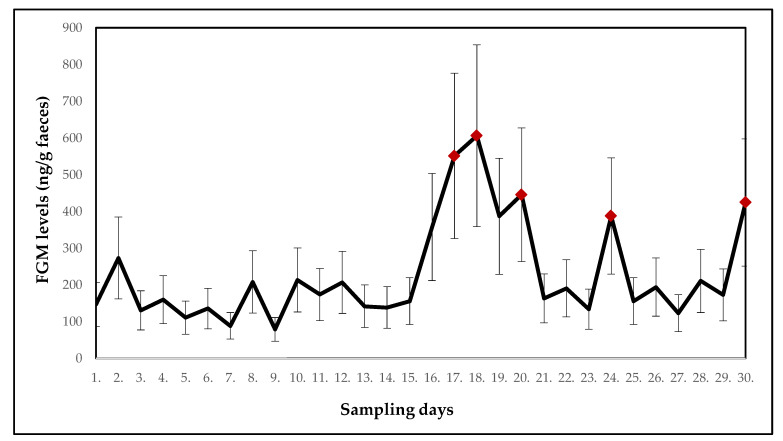
Mean concentrations of the faecal glucocorticoid metabolites (FGMs) in collared brown lemurs with significant increases compared to the first day of sampling indicated. Red points indicate a significant increase in FGM levels.

Fluctuations in the mean FGM levels in collared brown lemurs (*n* = 6) over a 30-day period of monitoring are shown in Figure 4. A statistically significant (*p* < 0.05) increase in the mean FGM levels (F_(29.145)_ = 2.1247) was found on days 17, 18, 20, 24 and 30.

Table 4 shows the mean FGM levels in collared brown lemurs and potentially stressful events for each day of monitoring. 

### 3.4. Species Comparisons

**Figure 5:**
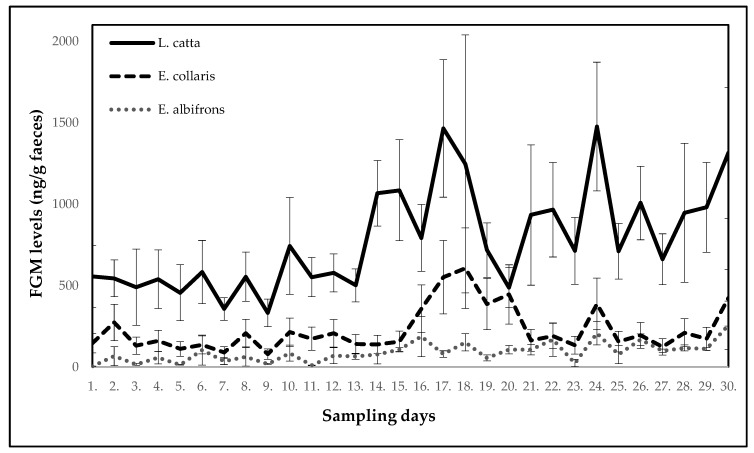
Comparison of glucocorticoid levels in three lemur species.

When comparing the FGM levels between all three lemur species, significant differences (*p* < 0.05) were observed. As shown in Figure 5, the highest levels were measured in ring-tailed lemurs, then in collared brown lemurs, and the lowest concentrations of FGMs were found in white-headed lemurs.

## 4. Discussion

Lemurs in captivity are exposed daily to potentially stressful situations. This is even more pronounced in the case of temporary housing facilities. In addition to the normal daily routine, which includes regular morning cleaning of all quarters and providing food twice a day, construction work was in progress in the monitored facility. Moreover, the observed lemurs were moved, and new animals arrived during the observation period. Such activities that are not carried out regularly are especially likely to cause stress to animals. In all lemur species, significantly increased FGM levels were found during the monitored period. The fluctuation of FGM levels could be caused by the recorded stressors, as elevated concentrations were often found 1 to 2 days after the events that deviated from the daily routine. While blood glucocorticoid concentrations increased shortly after exposure to an acute stressor, and in faeces, the increase of glucocorticoids resulting from stress was seen with a delay [7]. 

On the 13th and 14th day of the monitored period, all-day construction work was carried out near the room where the ring-tailed lemurs were housed. In the days after (14th and 15th day) a significant increase (*p* < 0.05) in the mean FGM levels was found. This increase could result from ongoing construction work as lemurs were exposed to increased noise and vibrations in their cages. The mean FGM levels also increased on day 17 (*p* < 0.01) and day 24 (*p* < 0.05). These increases could be related to relocations of two females with offspring (R3 and R4) into separate cages (16th and 23rd day). The separation was necessary due to agonistic behaviour observed in the females. Despite the females being familiar with each other from their original location, stress from the new environment could cause changes in their behaviour. Relocation can be perceived as a stressor in captive wild animals because they perceive all changes and new situations as a potential threat [28]. On day 23, one female (R1) was moved to a separate cage in the same room due to an injury. The injured female exhibited no significant increase in FGM levels, however, mean FGM levels in the group of ring-tailed lemurs increased. Finally, also on the last day of monitoring, a significant increase in the mean FGM values (*p* < 0.01) was observed. According to records, two giant otters (*Pteronura brasiliensis*) were relocated into the facility on the previous day which could be stressful for lemurs housed nearby. In the monitored period, relatively stable FGM levels were found on days which were not preceded by any exceptional events (daily routine was followed). Assumedly, the above-mentioned disturbances were responsible for the changes in FGM levels and they showed a consistent one-day delay before elevating FGM concentrations. White-headed lemurs were exposed to the same potentially stressful events as the ring-tailed lemurs. Construction work was in progress on day 13 and 14 near their enclosure, which could explain a significant increase (*p* < 0.05) in the mean FGM levels on days 15 and 16. Interestingly, there was a two-day delay before the FGMs elevated in white-headed lemurs in contrast with the one-day delay in ring-tailed lemurs following the same stressor. The significantly elevated levels of FGMs were found also in the last days of monitoring (day 26 to 29). The reason is unclear. The records did not show any deviations from the daily routine. However, the animals might have responded even to some minor changes not detected or perceived by the caretakers. According to Morgan and Tromborg [2], animals can respond to the inability to escape from human reach and the daily routine of cleaning the enclosure. Although routine is a repeated activity for a human being, an animal in captivity may perceive this situation differently and may be stressed. For example, primates have been shown to have high heart rates at the time of the keeper’s arrival at the enclosure due to routine cleaning [2]. Thus, for some more sensitive individuals, the presence of a person in their vicinity can be stressful, especially if they are in an unknown place [2,29].

In both, the ring-tailed lemurs and the white-headed lemurs, a significant (*p* < 0.05) increase in FGM levels was recorded following the construction work throughout the day in the yard of the facility where both species were housed. As Morgan and Tromborg [2] reported, excessive noise caused by, for example, noisy construction work, is stressful and affects not only the animal’s behaviour but also the levels of specific stress markers. In captive wild animals, acute and chronic stress caused by difficulties in coping with stressors such as public presence and noise, among others, can induce a significant increase in FGMs and repetitive pathological behaviours such as stereotypies [30,31].

Collared brown lemurs were housed in a different room than the other two lemur species. Thus, they could be affected by different potentially stressful events. In particular, these included necessary relocations due to the occurrence of agonistic behaviour in the group. In the monitored period, a significant increase (*p* < 0.05) in the mean FGM levels was found on days 17, 18, 20, 24 and 30, which could be caused by aggression and subsequent relocations. Mutual fights escalated especially during feeding. Aggressive behaviour has been proven to affect FGM levels, with dominant individuals having higher glucocorticoid levels [32,33,34]. However, also the movement itself or other changes in the group affect the level of the FGMs, as was demonstrated in the ring-tailed lemurs.

While the significant increase of FGMs in ring-tailed and white-headed lemurs was caused the next day after the arrival of giant otters, in collared brown lemurs no significant change was found in FGM levels following the arrival of Bennett’s wallabies (*Macropus rufogriseus*) to the same room. Furthermore, in collared brown lemurs there was no increase in the mean FGM levels in response to the construction work carried out on the 13th and 14th day of the observation, likely because the collared brown lemurs were located on the other side of the facility, thus no noises or vibrations from the construction work were recorded here.

During the monitored period, handling of animals was most often associated with their relocation to other cages due to agonistic behaviour. For relocation, capture and direct handling was necessary. That can be perceived as a strong stressor [24,28] with handling duration affecting the stress response and subsequently FGM levels [35]. However, it also depends on other factors, such as temperament of the individual and previous experience with the stressor. Considering their activity, it is generally recommended to capture lemurs in early morning hours and only by experienced caretakers [36,37]. The lemurs who were relocated in our study were captured by an experienced caretaker with the use of net and transferred to the new cage. Capture of animals is considered an acute stressor, but it can have longer lasting effects if it is carried out repeatedly in a short period [38]. According to Balcombe et al. [38], handling of animals is one of the most common husbandry practices but it results in variously strong stress responses and affects the activity of the immune system of animals. Hämäläinen et al. [24] measured the level of stress in grey mouse lemurs (*Microcebus murinus*) based on the determination of elevated levels of FGMs after capture. Although elevated levels have been recorded in some cases, routine capture has not had long-term consequences. The stress response in black howler monkeys (*Alouatta caraya*) was evaluated by Rangel-Negrín et al. [39]. The results showed that after capture, the levels of FGMs were affected. Similarly, Volfova et al. [18] recorded the increase of FGMs in response to handling in ring-tailed lemurs. Captive wild animals are expected to have a stronger response to handling stress than domesticated animals [40]. Given that handling is one of the primary stressors for captive wild animals, it is essential to monitor its potential negative impact on their welfare and avoid it as much as possible. 

The results show that all the three monitored lemur species respond during the observed period to specific stressful situations by increasing (*p* < 0.05) the FGM levels similarly within one to two days after the event. This delay from the stress event to GC excretion into faeces was confirmed not only in previous studies regarding handling of the ring-tailed lemurs and transportation of the black-and-white ruffed lemurs, but also in many other studies concerning primates [13,21,23,24,41]. Generally, the delay is 24 to 48 h in primates [22]. In white-headed lemurs, however, differences in response to potentially stressful situations were reported compared to ring-tailed lemurs. Increased average levels of the FGMs were found more frequently in white-headed lemurs than in ring-tailed lemurs. The results may indicate that this species responds more sensitively to changes in its surroundings. Nevertheless, these differences may also be due to the different temperament of the animals, as some studies confirm [6,7], as well as by the differences in the structure of the gastrointestinal tract in different lemurs species [7,42], which affects the amount of glucocorticoids secreted into the faeces [6]. It has been proven that there are differences in the metabolism of glucocorticoid hormones and their secretion into faeces between different species [6,43]. However, closely related species can be expected to have a similar or even equal representation of glucocorticoid hormone metabolites in their faeces and the time for which there is a demonstrable increase in the FGMs. 

The differences in the FGM concentrations in the individual species may be also due to different proportions of sex in the observed groups [44], because sex has a demonstrable impact on glucocorticoid levels, with females in oestrous, pregnant or lactating exhibiting elevated circulating glucocorticoids [6]. In our case, the ring-tailed lemur group consisted of eight females and one male, which is the standard group structure even in the wild. Three of these females had offspring and were lactating; the GC levels measured in their faeces thus increased the mean FGMs of the whole group. Similarly, Starling et al. [45] found elevated levels of the FGMs in lactating ring-tailed lemur females living in the wild. The group of white-headed lemurs, where the mean FGM levels were the lowest, consisted of two males. The group of collared brown lemurs consisted of four females and two males. The influence of sex on FGM levels was also confirmed by Arias et al. [46], who reported higher cortisol levels in female lama guanicoe (*Guanaco*) compared to males due to pregnancy. Dantzer et al. [47] found female American red squirrel (*Tamiasciurus hudsonicus*) also have elevated levels of FGMs during pregnancy. Furthermore, Carrera et al. [48] found higher glucocorticoid levels to be associated with low rank (compared to high rank) and first-time mothers (compared to multiparous mothers) in geladas (*Theropithecus gelada*).

The study has shown that some situations and activities related to captive housing can result in elevated levels of FGMs in lemurs. A similar pattern of changes in the FGM levels in response to potentially stressful situations was observed in the monitored lemurs. All three lemur species in our case showed a significant increase (*p* < 0.05) in the FGM levels within one, or at most two days after the stressor exposure. Total concentrations of the FGMs differed between the species, with the highest in ring-tailed lemurs, lower in collared brown lemurs and the lowest in white-headed lemurs. In white-headed lemurs, much lower FGM levels were found in comparison with the other two lemur species, which could be interpreted as a species difference. However, it is also possible that their low levels of FGMs corresponded to the fact that as the only one their group was not divided during the monitored period, and the males were used to each other. For social species, their mutual dependence is high [49]. Furthermore, it was also the only male group. The absence of females could also lead to an overall lower level of FGMs. In contrast, the highest glucocorticoid concentrations in ring-tailed lemurs could have been caused by the presence of lactating females in the group. Furthermore, the individual animal’s temperament or social status may affect the absolute FGM levels. 

Limitations of the study included lack of baseline data, varying time of arrival of individual animals to the facility (and thus varying period of acclimatization or lack thereof before the commencement of the study), different number of animals and varying sex ratio in the monitored groups. However, it was not possible to experimentally design and control the structure of the monitored groups and the conditions of observation. 

## 5. Conclusions

Stress is currently one of the most serious problems in rearing endangered animal species in zoos and other facilities around the world. For their preservation and successful breeding, it is therefore of the utmost importance to avoid excessive stress and to provide the most suitable conditions for their life in captivity. In order for the housing conditions to be properly assessed, it is necessary to determine the impact of the factors to which captive lemurs are exposed. This study shows fluctuations in FGM levels in three lemur species likely resulting from changes in the structure of a group with relocation of individuals, construction work in the immediate vicinity of animals or placing another animal in quarters adjacent to the observed individuals. The results suggest that some lemur species may be more or less sensitive to such disturbances. However, FGM concentrations had a similar pattern (corresponding to the occurrence of potentially stressful events) during 30 days of monitoring in all three species of *Lemuridae* suggesting that closely related animal species respond to stress load similarly. A significant increase in the FGM levels was found within one, or at most two days after the exposure to a stressor. 

## Figures and Tables

**Table 1:** Lemurs observed in a temporary housing facility.

Species	Animal	Sex	Number of Offspring	Birth	Original Zoo	Arrival to the Facility
Ring-tailed lemur	R1	F		unknown	Zoo Wroclaw	1 month before observation
	R2	F		unknown	Zoo Wroclaw	
	R3	F	2	March 2002	Zoo Wroclaw	
	R4	F	1	June 2009	Zoo Wroclaw	
	R5	F		March 2014	zoo Duisburg	3 weeks before observation
	R6	F	1	March 2012	zoo Duisburg	
	R7	F		March 2014	zoo Bernburg	
	R8	M		unknown	Zoo Wroclaw	
	R9	F		March 2016	Hamerton zoo park	first day of observation
White-headed lemur	W1	M		April 2011	Olmense zoo	2 weeks before observation
	W2	M		April 2012	Olmense zoo	
Collared brown lemur	C1	M		April 2015	Hamerton zoo park	first day of observation
	C2	M		April 2015	Hamerton zoo park	
	C3	F		March 2015	Hamerton zoo park	
	C4	F		unknown	Hamerton zoo park	
	C5	F		April 2016	Hamerton zoo park	
	C6	F		April 2015	Hamerton zoo park	

**Table 2:** Mean concentrations of the faecal glucocorticoid metabolites (FGMs) in ring-tailed lemurs and potentially stressful events for each day of monitoring.

Ring-Tailed Lemur (*Lemur catta*)										
**Day**	1	2	3	4	5	6	7	8	9	10
**Mean ± SE (ng/g faeces)**	556.5 ± 189.7	544.3 ± 113.0	490.1 ± 233.8	538.5 ± 180.0	456.1 ± 171.7	582.9 ± 194.2	355.7 ± 69.7	554.2 ± 151.6	332.0 ± 84.7	743.5 ± 297.3
***p*-value**	-	0.099	0.263	0.491	0.646	0.954	0.344	0.774	0.160	0.456
**Potential stressor**	DR	DR	DR	DR	DR	DR	DR	DR	DR	DR
**Day**	11	12	13	14	15	16	17	18	19	20
**Mean ± SE (ng/g faeces)**	552.2 ± 120.3	577.7 ± 116.9	501.1 ± 101.6	1065.9 ± 201.4	1085.2 ± 310.0	792.1 ± 205.2	1464.3 ± 421.9	1246.7 ± 791.8	717.4 ± 166.7	488.0 ± 122.4
***p*-value**	1.000	0.886	0.863	0.037	0.097	0.197	0.006	0.796	0.547	0.752
**Potential stressor**	DR	DR	construction work	construction work	DR	relocation of two females with offspring	DR	DR	DR	DR
**Day**	21	22	23	24	25	26	27	28	29	30
**Mean ± SE (ng/g faeces)**	933.0 ± 430.9	965.5 ± 290.3	712.5 ± 204.8	1476.2 ± 395.2	710.4 ± 170.3	1006.6 ± 225.8	661.3 ± 156.4	946.9 ± 426.6	979.8 ± 275.5	1314.0 ± 403.2
***p*-value**	0.863	0.178	0.863	0.018	0.841	0.136	0.605	0.752	0.109	0.010
**Potential stressor**	DR	DR	relocation of one female	DR	DR	DR	DR	DR	relocation of giant otters	DR

DR = daily routine

**Table 3:** Mean concentrations of the faecal glucocorticoid metabolites (FGMs) in white-headed lemurs and potentially stressful events for each day of monitoring.

White-Headed Lemur (*Eulemur albifrons*)										
**Day**	1	2	3	4	5	6	7	8	9	10
**Mean ± SE (ng/g faeces)**	3.7 ± 1.0	66.1 ± 57.9	16.1 ± 5.6	56.5 ± 37.3	13.1 ± 2.1	103.5 ± 92.5	36.2 ± 21.6	62.7 ± 57.2	18.8 ± 3.2	84.7 ± 49.2
***p*-value**	-	0.178	0.602	0.246	0.672	0.071	0.301	0.141	0.365	0.041
**Potential stressor**	DR	DR	DR	DR	DR	DR	DR	DR	DR	DR
**Day**	11	12	13	14	15	16	17	18	19	20
**Mean ± SE (ng/g faeces)**	9.8 ± 1.5	68.8 ± 49.2	63.7 ± 16.1	76.6 ± 57.3	106.8 ± 11.7	187.1 ± 123.6	79.3 ± 19.9	151.7 ± 52.9	55.7 ± 18.6	106.2 ± 25.6
***p*-value**	0.649	0.125	0.168	0.054	0.021	0.013	0.086	0.010	0.111	0.017
**Potential stressor**	DR	DR	construction work	construction work	DR	relocation of three females	DR	DR	DR	DR
**Day**	21	22	23	24	25	26	27	28	29	30
**Mean ± SE (ng/g faeces)**	106.2 ± 32.2	168.3 ± 102.3	23.9 ± 23.7	207.5 ± 71.9	76.3 ± 55.7	167.6 ± 35.9	98.7 ± 10.7	117.3 ± 18.3	110.3 ± 8.5	256.0 ± 148.2
***p*-value**	0.016	0.013	0.625	0.002	0.058	0.002	0.033	0.008	0.013	0.005
**Potential stressor**	DR	DR	relocation of one female	DR	DR	DR	DR	DR	relocation of giant otters	DR

DR = daily routine

**Table 4:** Mean concentrations of the faecal glucocorticoid metabolites (FGMs) in collared brown lemurs and potentially stressful events for each day of monitoring.

Collared Brown Lemur (*Eulemur collaris*)										
**Day**	1	2	3	4	5	6	7	8	9	10
**Mean ± SE (ng/g faeces)**	146.7 ± 59.9	273.4 ± 111.6	130.6 ± 53.3	159.5 ± 65.1	110.5 ± 45.1	135.4 ± 55.3	88.5 ± 36.1	207.9 ± 84.9	78.8 ± 32.2	213.3 ± 87.1
***p*-value**	-	0.213	0.413	0.803	0.545	0.854	0.393	1.000	0.177	0.721
**Potential stressor**	DR	DR	relocation of wallabies	DR	DR	DR	DR	DR	DR	DR
**Day**	11	12	13	14	15	16	17	18	19	20
**Mean ± SE (ng/g faeces)**	173.6 ± 70.9	206.8 ± 84.4	141.9 ± 57.9	138.5 ± 56.5	156.0 ± 63.7	357.6 ± 146.0	551.1 ± 225.0	606.1 ± 247.4	386.9 ± 158.0	445.5 ± 181.9
***p*-value**	0.803	0.915	0.521	0.830	0.521	0.695	0.010	0.034	0.240	0.047
**Potential stressor**	DR	DR	DR	DR	DR	relocation of two males	DR	relocation of one female	DR	DR
**Day**	21	22	23	24	25	26	27	28	29	30
**Mean ± SE (ng/g faeces)**	163.1 ± 66.6	190.8 ± 77.9	133.9 ± 54.7	387.4 ± 158.2	155.7 ± 63.6	194.0 ± 79.2	122.9 ± 50.2	210.5 ± 85.9	172.8 ± 70.5	424.2 ± 173.2
***p*-value**	0.775	0.498	0.721	0.028	0.873	0.748	0.775	0.618	0.901	0.015
**Potential stressor**	DR	DR	relocation of one male	DR	DR	DR	DR	DR	relocation of two males and one female	DR

DR = daily routine
